# Home birth attendants in low income countries: who are they and what do they do?

**DOI:** 10.1186/1471-2393-12-34

**Published:** 2012-05-14

**Authors:** Ana Garces, Elizabeth M McClure, Elwyn Chomba, Archana Patel, Omrana Pasha, Antoinette Tshefu, Fabian Esamai, Shivaprasad Goudar, Adrien Lokangaka, K Michael Hambidge, Linda L Wright, Marion Koso-Thomas, Carl Bose, Waldemar A Carlo, Edward A Liechty, Patricia L Hibberd, Sherri Bucher, Ryan Whitworth, Robert L Goldenberg

**Affiliations:** 1 IMSALUD, Guatemala City, Guatemala; 2 Research Triangle Institute, Durham, NC, USA; 3 Department of Pediatrics, University Teaching Hospital, Lusaka, Zambia; 4 Indira Ghandi College of Medicine, Nagpur, India; 5 Department of Community Health Sciences, Aga Khan University, Karachi, Pakistan; 6 Kinshasa School of Public Health, Kinshasa, Democratic Republic of Congo; 7 Department of Pediatrics, Moi University, Eldoret, Kenya; 8 Department of Medical Education, Jawaharlal Nehru Medical College, Belgaum, India; 9 Department of Pediatric Nutrition, Denver University School of Public Health, Denver, CO, USA; 10 Center for Research for Mothers and Children, Eunice Kennedy Shriver National Institute of Child Health and Human Development, Bethesda, MD, USA; 11 Department of Pediatrics University of North Carolina at Chapel Hill, Chapel Hill, NC, USA; 12 University of Alabama at Birmingham, Birmingham, AL, USA; 13 Department of Pediatrics, Indiana University, Indianapolis, IN, USA; 14 Massachusetts General Hospital, Boston, MA, USA; 15 Department Obstetrics and Gynecology, Columbia University, NY, USA

**Keywords:** Home births, Traditional birth attendants, Perinatal mortality

## Abstract

**Background:**

Nearly half the world’s babies are born at home. We sought to evaluate the training, knowledge, skills, and access to medical equipment and testing for home birth attendants across 7 international sites.

**Methods:**

Face-to-face interviews were done by trained interviewers to assess level of training, knowledge and practices regarding care during the antenatal, intrapartum and postpartum periods. The survey was administered to a sample of birth attendants conducting home or out-of-facility deliveries in 7 sites in 6 countries (India, Pakistan, Guatemala, Democratic Republic of the Congo, Kenya and Zambia).

**Results:**

A total of 1226 home birth attendants were surveyed. Less than half the birth attendants were literate. Eighty percent had one month or less of formal training. Most home birth attendants did not have basic equipment (e.g., blood pressure apparatus, stethoscope, infant bag and mask manual resuscitator). Reporting of births and maternal and neonatal deaths to government agencies was low. Indian auxilliary nurse midwives, who perform some home but mainly clinic births, were far better trained and differed in many characteristics from the birth attendants who only performed deliveries at home.

**Conclusions:**

Home birth attendants in low-income countries were often illiterate, could not read numbers and had little formal training. Most had few of the skills or access to tests, medications and equipment that are necessary to reduce maternal, fetal or neonatal mortality.

## Background

Of the world’s estimated annual 130 million births, nearly half occur at home [1]. Outcomes of these births, including maternal, fetal and neonatal mortality, are reported to be considerably worse than those occurring in a hospital [1,2]. Attendants at these births may be physicians, trained nurses or midwives, other trained professionals, family members, friends or traditional birth attendants.

To date, policy makers have little valid information on which to base policies about home birth attendants’ (HBA) roles, both in terms of managing childbirth and in implementing effective referral systems [3]. Assessing HBAs’ demographic data, their skills in reading and the use of numbers, level of training, and birth attendants’ relationship with other health services could be important in defining their potential role in the care of women and infants, and for developing the best strategies to improve their practices.

Thus, three primary reasons underlie the decision to undertake the survey reported here: 1) a significant proportion of the births world-wide occur at home, 2) birth attendant skill level and access to diagnostic and therapeutic interventions are likely to impact directly on maternal and perinatal mortality, and 3) there is a general lack of information on the knowledge, training, skills and practices of those performing home births. To better understand the characteristics of those performing home births, we surveyed a sample of non-physicians performing home births in seven developing country sites. We purposely chose not to classify those performing home deliveries *a priori* into arbitrary categories (nurses, midwives, traditional birth attendants), but acquired the same information on all home birth providers in order to capture data on the full range of HBAs practicing in the communities studied.

## Methods

The Global Network for Women’s and Children’s Health Research (Global Network), funded by the *Eunice Kennedy Shriver* National Institute for Child and Human Development, is comprised of 8 US university/international institution partnerships that perform collaborative research to improve maternal, fetal, neonatal and childhood health outcomes. Sites are located in the Western Province of Kenya, Kafue District of Zambia, The Equateur Province of the Democratic Republic of the Congo (DRC), the Thatta District of the Sindh Province in Pakistan, India (Karnataka State, Belgaum and Nagpur), Chimaltenango Provence Guatemala and Argentina. (The Argentina site has no HBAs and therefore did not participate in this survey.) We have identified 106 population based geographic areas (clusters) with approximately 500 births per year in each of the clusters. In each cluster, project staff attempt to register all women by mid-pregnancy, and collect multiple pregnancy outcomes on all deliveries, including delivery attendant and site of delivery (i.e., health facility-based vs. non-facility based). Throughout the paper we use the term home birth attendant (HBA) to describe attendants performing home - or out of facility - births.

Using these birth registry data, the data coordinating center (Research Triangle Institute [RTI]) identified all birth attendants in each cluster conducting deliveries outside of health facilities. Any active non-physician birth attendant (e.g., one who had conducted at least 5 deliveries per year) currently delivering babies outside of health facilities, regardless of their training, was eligible to be included in the survey. Each site was expected to interview a minimum of 100 home/out-of-facility birth attendants. In order to reduce selection bias among those interviewed, from those eligible, RTI randomly selected 100 HBAs for interview. Some of the sites had the capacity to do additional interviews and for these sites, RTI randomly selected a second hundred for interview. The Guatemala site attempted to interview all HBAs practicing in their study cluster. The data in the tables and figures are presented as a percentage of HBAs interviewed by site. In the total column, the results are presented as a percent of all HBAs interviewed, unadjusted for site. In undertaking the survey, we learned that both Indian sites had a class of birth attendants working predominantly in clinics who also did some home deliveries - auxilliary nurse midwives (ANMs). Because these birth attendants were so different from the more traditional HBAs surveyed at the other sites, as well as from the more traditional HBAs surveyed at the Nagpur site, we present their data separately. Thus, the final column presents results for the Indian ANMs, who perform deliveries both at home and in community clinics.

Each site obtained institutional ethics review board approval, including IMSALUD (Guatemala), Indira Ghandi College of Medicine (Nagpur, India), Kinshasa School of Public (Kinshasa, DRC), University Teaching Hospital (Lusaka, Zambia), Moi University (Eldoret, Kenya), JN Medical College (Belgaum, India), Aga Khan University (Karachi, Pakistan) and Research Triangle Institute on behalf of the Data Coordinating Center prior to implementation of the study. Birth attendants provided informed written (or, if necessary, oral) consent to participate in this study of their educational and training background, as well as their current birth practices. The survey was administered as a face-to-face structured questionnaire which took approximately 40 minutes. Surveys were checked for completeness and the data entered on-site by each participating research unit. Data were transmitted to RTI for central editing, and analyzed using SAS (v.9.2). Descriptive analyses were performed to calculate the statistics and ranges for each variable.

## Results

Table 1 presents data for each of the participating sites regarding birth location and type of birth attendant. One site interviewed fewer, and a number of sites elected to interview more than the planned sample size of 100 CBAs per site. The proportion of births occurring at home ranged from 14% in Nagpur, India to 74% in the Equateur Province of DRC. Home birth attendants performed between 8%–75% of deliveries (Belgaum, India and DRC, respectively), with high rates of HBA-attended births also reported for Guatemala. In two sites, many of the births were attended by family members or were unattended (Zambia 20% and Kenya 13%). In both Indian sites, more than 70% of births occurred in health centers or hospitals and these births were generally attended by nurses, ANMs, or physicians.

**Table 1:** Characteristics of births at study sites (2010)

	DRC*	**Zambia**	**Kenya**	**Guatemala**	**Pakistan**	**Nagpur, India**	**Belgaum, India**
**Number births**	14,306	16,352	18,319	10,590	34,379	15,022	34,509
**Birth location**							
Home (%)	74.4	52.7	64.7	71.0	56.6	14.0	16.3
Health ctr(%)	23.6	41.7	9.4	3.1	21.9	25.7	23.2
Hospital(%)	1.0	5.6	25.9	25.9	21.1	53.5	60.3
**Birth attendant**							
Family/home (%)	3.0	20.1	12.8	0.2	2.0	3.9	7.1
HBA** (%)	75.3	34.2	51.4	70.6	54.1	9.6	8.2
Nurse/midwife (%)	21.6	43.1	34.1	1.4	23.8	33.1	29.9
Physician (%)	0.1	2.6	1.7	27.8	20.0	53.5	37.9

*DRC data reported in 2008.

**Home birth attendant.

A total of 1,226 attendants who performed home births (including the Indian ANMs) were surveyed at the 7 sites. In Table 2, the data for the untrained HBAs are presented for each site, and then summarized as a total in the next to last column. In the last column, the data on the ANMs from both Indian sites combined are presented. Nearly all HBAs were female. For the HBAs, the median age of all surveyed was 53 years. The median number of years of school attendance was 0 and across the sites, only 8% of the HBAs surveyed had obtained any degree for school completion. Table 2 also presents data on resources available to the HBAs. Overall, only 54% had electricity in their homes, and only 29% had an indoor toilet. 45% of the HBAs had access to a cell phone, 30% owned a bicycle and only 19% had access to motorized transportation. The Indian ANMs were younger (median age of 37), had far more schooling and were more likely to have school degrees, (67%) and were also more likely to have electricity (99%) and toilets (92%) in their home and have cell phones (92%).

**Table 2:** Birth attendant socio-economic and demographic characteristics

	**Home Birth Attendants**							**Auxilliary Nurse Midwives**
**Characteristics**	**DRC**	**Zambia**	**Kenya**	**Guatemala**	**Pakistan**	**Nagpur, India**	**Total**	**India**
**Number enrolled**	102	208	100	434	105	103	1052	174
Female (%)	99.0	100	100	100	97.1	100	99.6	94.8
Age, yrsMedian (min-max)	50 (28-78)	49 (25-70)	57 (25-81)	56 (23-92)	55 (33-85)	50 (27-77)	53 (23- 92)	37 (22- 73)
Schooling, yrsMedian (min-max)	0 (0-10)	7 (0-20)	1 (0-12)	0 (0-12)	0 (0-10)	1 (0-21)	0 (0-21)	12 (0-20)
School(degree obtained) (%)	1.0	17.8	0	11.1	1.0	0	8.3	67.2
Cook on gas stove (%)	1.0	4.3	2.0	8.1	17.1	7.8	6.9	48.3
Electricity in home (%)	0	20.2	3.0	85.5	60.0	88.3	54.2	99.4
Indoor toilet in home (%)	1.0	14.4	2.0	44.0	43.8	35.0	29.1	92.0
Own cell phone (%)	0	77.9	39.0	55.3	18.1	14.6	45.2	92.5
Own bicycle (%)	33.3	36.5	65.0	27.0	21.0	1.9	30.0	49.4
Access to Motorbike or other motorized vehicle (%)	0	10.1	53.0	20.5	32.4	1.0	18.8	7.5

Table 3 provides data on HBA training, experience and skills. Although there was considerable variation, 87% of the traditional HBAs across all sites had one month or less of organized training. Overall, 30% of HBAs were literate, although literacy levels varied substantially across the sites. In Pakistan, only 3% of the HBAs were literate. Across all the sites, the ability to write ranged from 2% in Pakistan to as high as 41% in Zambia. The ability to read and write numbers had similar variability, although on average, more HBAs claimed ability to write numbers than to write words. There was also wide variability among the sites in the ability of HBAs to use a calendar and tell time. Few HBAs were able to count a heartbeat with a stethoscope. In contrast, 64% of the Indian ANMs had more than one year of formal training, 73% were literate and 97% or more could read and write numbers, use a calendar, tell time and count a heartbeat with a stethoscope.

**Table 3:** Medical Training and Skills*

	**Home Birth Attendants**							**Auxilliary Nurse Midwives**
	**DRC**	**Zambia**	**Kenya**	**Guatemala**	**Pakistan**	**Nagpur, India**	**Total**	**India**
**Number enrolled**	102	208	100	434	105	103	1052	174
**Training**								
None (%)	38.2	9.6	46.0	1.2	50.5	7.8	16.3	0.6
< 1 mo (%)	60.8	62.5	53.0	85.5	45.7	74.8	70.4	28.7
1-12 mos (%)	1.0	16.3	0	12.9	3.8	16.5	10.6	6.3
>1 yr (%)	0	11.5	1.0	0.5	0	1.0	2.7	64.4
Experience yrs, median, (min-max)	15 (1-38)	7 (1-39)	16 (0-58)	18 (0-60)	8 (0-40)	20 (2-57)	15 (0-60)	10 (1-46)
Literate (%)	12.7	41.8	38.0	38.7	2.9	7.8	30.1	73.0
Can Write (%)	5.9	40.9	38.0	38.7	1.9	7.8	29.2	70.7
Read numbers (%)	24.5	94.2	62.0	50.5	16.2	62.1	55.4	98.3
Write numbers (%)	24.5	91.3	54.0	47.2	3.8	50.5	50.4	96.6
Use Calendar (%)	23.5	95.2	62.0	48.2	8.6	57.3	53.3	97.1
Tell time (%)	24.5	97.6	61.0	63.4	26.7	68.0	62.9	98.3
Count heart beat with stethoscope (%)	1.0	23.6	0.0	22.1	1.0	3.9	14.4	96.6

*Results presented at percentages, unless otherwise noted.

**Figure 1:**
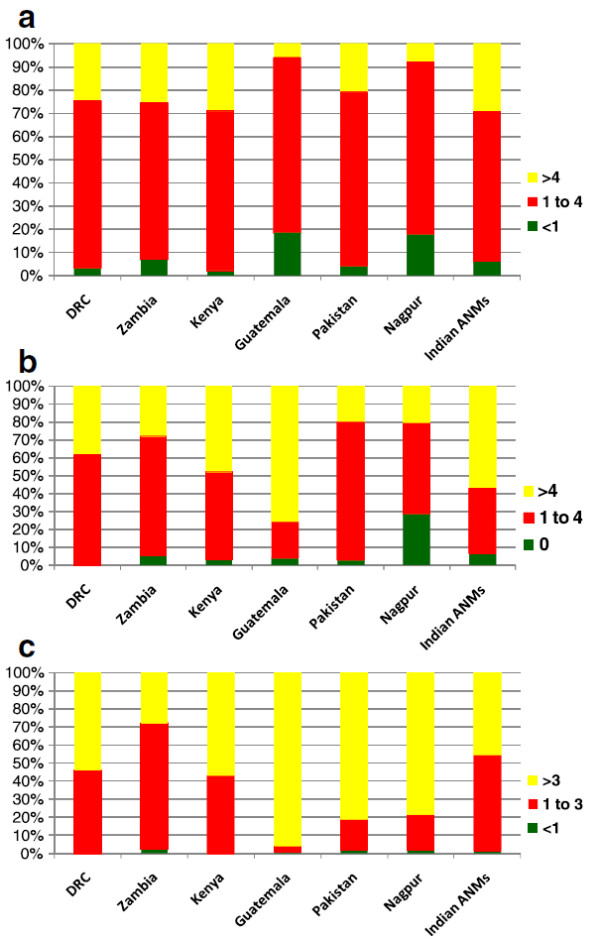
a) Average number of deliveries per month, b) Average number of prenatal care visits, c) Average number of prostnatal care visits.

Figure 1 (a, b and c) presents data on practice volume for the HBAs and ANMs. In most sites, both the HBAs and ANMs reported attending between one to four deliveries per month. In all sites, nearly all HBAs and ANMs reported providing some antepartum and post-partum visits.

Table 4 presents data on HBA practices by site. In most sites, 70% or more of the HBAs reported using clean birth kits for all deliveries. Exceptions included Zambia (61%) and the DRC (2%). Only 8% of HBAs reported that they routinely take blood pressures and even fewer (3%) ever repair vaginal lacerations. Limited numbers of HBAs reported performing episiotomies (4%). Nearly all HBAs in all sites report that they always dried the baby, but cleaning the mouth with gauze and using a suction bulb was variable across sites. The ANMs almost always took blood pressures and 65% repaired lacerations. Spanking or shaking a newborn to stimulate respiration, an out-of-date neonatal resuscitation practice now discouraged by many authorities, was variably performed but commonly practiced across most of the sites, including the Indian ANMs.

**Table 4:** Birth Attendant Practices (%)

	**Home Birth Attendants**							**Auxilliary Nurse Midwives**
**Practice**	**DRC**	**Zambia**	**Kenya**	**Guatemala**	**Pakistan**	**Nagpur, India**	**Total**	**India**
**Number enrolled**	102	208	100	434	105	103	1052	174
Always use clean birth kit	2.0	61.1	100	92.9	77.1	59.2	73.6	98.3
Always take blood pressure	1.0	19.2	0.0	8.3	0.0	2.9	7.6	93.7
Ever repair vaginal laceration	1.0	8.2	4.0	2.3	0.0	1.0	3.1	65.3
Ever perform episiotomy	0.0	7.2	22.0	0.7	0.0	1.0	3.9	50.3
Always use bulb suction	69.9	12.5	2.0	85.7	8.6	16.5	47.2	55.8
Always clean mouth with gauze	61.8	79.3	98.0	93.8	98.1	93.2	88.6	97.7
Always dry baby	95.1	98.6	97.0	99.1	93.3	82.5	96.2	91.2
Always spank or shake baby to stimulate breathing	64.4	51.0	14.0	56.9	51.4	66.0	52.7	51.5

Survey respondents were questioned about the availability of specific laboratory tests and equipment (Table 5). In general, HBAs reported that stethoscopes, fetoscopes, blood pressure cuffs, fundal height tapes, watches, thermometers, and bag and masks for newborn resuscitation were not routinely available to them. The HBAs’ access to laboratory tests across the sites was also generally limited, with the exception of the Kenyan site, specifically for a number of infectious diseases. In Kenya, the likely explanation for the high access to tests by HBAs is their provision by a local NGO. The Kenyan Government does not allow HBAs to order these tests themselves. For virtually every type of equipment and laboratory test, the Indian ANMs had substantially greater access and availability than the HBA’s.

**Table 5:** Access to laboratory tests and equipment (%)

	**Home Birth Attendants**							**Auxilliary Nurse Midwives**
**Characteristic**	**DRC**	**Zambia**	**Kenya**	**Guatemala**	**Pakistan**	**Nagpur**	**Total**	**India**
**Number enrolled**	102	208	100	434	105	103	1052	174
**Equipment**								
Clean birth kit	2.0	61.1	100	92.9	77.1	59.2	73.6	98.3
Maternal scale	3.9	48.1	4.0	3.5	1.9	21.4	14.0	93.6
Stethoscope	1.0	24.0	0.0	42.4	1.10	3.9	22.8	98.3
Blood pressure cuff	1.0	13.5	0.0	15.4	0.0	2.9	9.4	94.8
Fetoscope	33.3	27.9	14.0	24.7	8.6	2.9	21.4	72.9
Watch	11.8	67.3	25.0	46.8	17.1	35.0	41.3	97.7
Bag/mask	7.8	57.2	0.0	65.4	36.2	4.9	43.2	97.1
Thermometer	1.0	14.9	0.0	19.1	2.9	2.9	11.5	94.2
Fundal height tape	8.8	26.0	0.0	20.3	3.8	2.9	15.0	65.9
**Laboratory tests**								
Urine protein	0.0	4.3	27.0	9.2	20.0	1.0	9.3	58.6
Urine bacteria	4.9	3.8	69.0	24.0	3.8	0	18.1	33.3
Urine sugar	1.0	4.3	37.0	15.7	3.8	1.0	11.4	63.8
Fecal parasites	15.7	1.9	60.0	30.0	0.0	0	20.0	10.9
Anemia tests	13.7	5.3	86.0	30.4	33.3	5.8	27.0	64.4
HIV	12.7	18.3	91.0	46.1	1.0	3.9	33.0	90.2
Hepatitis	0.0	2.4	8.0	12.7	10.5	0	7.5	44.3
Syphilis	1.0	13.5	14.0	9.2	2.9	2.9	8.5	53.4
Malaria	14.7	13.5	71.0	0.7	18.1	3.9	13.3	62.6

**Figure 2:**
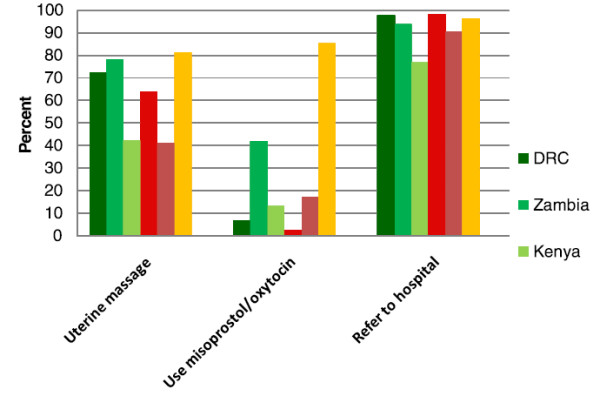
Maternal bleeding care practices.

Treatment of maternal bleeding by the HBAs is of special interest (Figure 2). Uterine massage - which is widely recommended as a first-line treatment for hemorrhage from an atonic uterus and should be within the scope of HBAs’ ability- was variably practiced across the sites, but was used by 80% of the Indian ANMs. The use of uterotonics such as oxytocin and misoprostol was low, especially in DRC, Kenya, Guatemala and Pakistan. The high use of uterotonics by the Indian ANMs is partly explained by the recent completion of a trial of misoprostol for post-partum hemorrhage at the Belgaum site. Most HBAs and ANMs across all sites reported that they nearly always referred women who were bleeding. For this study, we did not attempt to ascertain how frequently these referrals resulted in patient movement to an appropriate facility.

A number of additional questions addressed referral and reporting practices (Table 6). At all sites, more than 75% of the HBAs stated that they could make referrals, and similar numbers claimed to having made a referral. However, the proportion who had ever visited a referral center varied. For example, in Guatemala, less than half the HBAs had ever visited their referral center. When asked about maintaining a written birth log, the response of the HBAs was highly variable as well, with maintenance of a birth log reported by only 3% of Pakistani HBAs compared to 95% of Zambian HBAs. (Zambian respondents interpreted this question to indicate reporting all births to a study coordinator; however, other than for an ongoing study, virtually none of the Zambian HBAs kept an independent log of deliveries.) There were similar wide variations in reporting of births and deaths; no Pakistani HBA claiming to report these to health authorities while 94% of the Indian ANMs kept a birth log and virtually all reported all births, stillbirths and maternal deaths.

**Table 6:** Referral and reporting practices (%)

	**Home Birth Attendants**							**Auxilliary Nurse Midwives**
**Practice**	**DRC**	**Zambia**	**Kenya**	**Guatemala**	**Pakistan**	**Nagpur,** **India**	**Total**	**India**
**Number enrolled**	102	208	100	434	105	103	1052	174
Can make referrals	100.0	97.1	94.9	81.8	78.1	81.6	87.4	98.3
Ever made referral	97.1	95.1	96.8	91.3	98.8	97.7	94.6	99.4
Ever visited referral facility	99.0	98.6	75.0	42.9	90.5	78.6	70.6	86.7
Maintain birth log	80.4	94.7	66.0	42.3	2.9	6.9	51.3	93.6
Report births	96.1	98.6	43.0	84.8	0.0	64.1	74.1	99.4
Report stillbirths	80.4	88.5	25.0	83.6	0.0	64.1	68.4	100
Report maternal death	2.9	88.0	19.0	85.2	0.0	65.0	61.0	100

## Discussion

World-wide, each year, some 60 million births occur at home, and the pregnancy outcomes appear considerably worse than births that occur in a medical facility [1]. Much is still unknown about the attendants at these home births, particularly in regards to their training, delivery practices, access to medical equipment and testing, and their interaction with the formal health care system [4-9]. Prior investigations have generally been small pilot studies, conducted in limited areas [7-9]. Because these HBAs appear to play such a crucial role in pregnancy outcomes in many developing countries, we felt it important to obtain more detailed information about HBA knowledge, training, and delivery practices. The Global Network for Women’s and Children’s Health Research is a multi-site international consortium of research programs and birth registries which operates in regions of the world where maternal-newborn mortality rates are the highest. Thus, its members were well suited to carry-out this study.

This survey was conducted in geographic areas in which the NICHD Global Network has been conducting research for about 10 years [10-12]. This history suggests that in all likelihood, the results of the HBAs in this study demonstrated a *higher* level of baseline training and practices than if the survey had been conducted in adjacent, non-Global Network areas where HBA training and research studies had not occurred. For example, one clear difference noted between the DRC with almost no clean birth kit use and other participating sites with active use of clean birthing kits was likely explained by the fact that, because there were no current, ongoing Global Network studies in the area of the DRC where the HBA survey was conducted, clean birthing kits were not generally available. The high use of uterotonics in the Belgaum site was likely explained by a recent misoprostol research study conducted at that site.

The Indian ANMs, who conduct some home births, are different from the traditional HBAs in many ways. They generally have had a number of years of elementary and high school education before entering a Ministry of Health training program lasting a minimum of 18 months [13]. The ultimate goal is for them to perform deliveries in clinics throughout India. Therefore, even though they perform home births, we elected to summarize their data separately from the more traditional home birth attendants.

Focusing on the traditional HBAs, a number of conclusions can be drawn. The HBAs are generally older women with little schooling and are generally poor. Most do not have an indoor toilet, only 54% have electricity, most lack a gas or electric stove for cooking and less than half have access to a cell phone or some sort of transportation. Only about 30% can read or write and many cannot read numbers, tell time, or use a calendar. Very few have had more than a month of professional training. Compared to the required level of training for birth attendants in most developed countries, which generally ranges from three to more than 10 years post high school, this is a very low level of training.

In nearly all sites, the HBAs performed 1 to 4 deliveries per month. This relatively low range is likely explained in part by the often wide dispersion of communities geographically, and the lack of transportation available to many home birth attendants, restricting their potential clients to those within walking distance. This low number of deliveries means that most HBAs will experience a life threatening emergency only occasionally. The skills necessary to manage those emergencies, even if previously taught and mastered, often are forgotten because of disuse [14]. For example, it is estimated that only 3% of newborns require bag and mask resuscitation [15]. For a HBA delivering 40 babies a year, that skill will need to be applied perhaps once a year, and when taught, will likely soon be forgotten. In our experience from a previous study, many HBAs who had been provided a bag and mask and taught how to use it, no longer had this equipment in their possession a year after the study was over. (Personal observation RLG, EMM, CB) [10].

With few exceptions, HBAs had limited access to medical testing and equipment, and additionally, limited training to carry these out adequately. Testing that is standard practice in most high and middle income countries, such as measuring blood pressure or testing for anemia were available to less than half of them. Tests for infectious diseases, with the exception of HIV at some sites, were only rarely available. Few of the HBAs had a stethoscope or could repair a vaginal laceration. There were also practices employed, such as shaking or spanking the baby, that are generally viewed as ineffective and potentially harmful to newborn health. In the face of hemorrhage, less than half the HBAs reported the use of uterine massage, an important birth management practice that should be within the scope of home birth attendants’ delivery skills [16]. There are, however, some generally recommended practices such as drying the baby, clearing mucus from the baby’s mouth, and teaching exclusive breast feeding that are routinely used by the home birth attendants.

Most HBAs claimed to refer women with medical problems, particularly hemorrhage. Nevertheless, in several of the sites, nearly half of the home birth attendants also reported having never visited their referral hospital. Also the percentage of the women referred by home birth attendants who actually arrived at the hospital is unknown. From other unpublished data collected in these sites (RLG, EMM), we know that the actual rate of referral in these areas from home to any health facility is very low.

This study had several strengths. Our sample included a large number of HBAs from sites in six countries. The questionnaire covered a wide range of variables, including: demographics, practice capabilities, diagnostic testing, equipment and reporting/referral practices. Potential weaknesses include the fact that the study was carried out in areas where a number of research projects involving HBA training have been performed, and thus the results may not be representative of all HBAs in those countries, and may *overestimate* the HBA knowledge and/or skills compared to other regions. Also, the data presented are derived from self-reported surveys, and did not involve actual observation. Nevertheless, we believe this is one of the most comprehensive multi-country studies to evaluate skills and practices of a large sample of practicing HBAs.

We believe the information derived from this study will be useful to health care providers and policy makers as they try to improve pregnancy outcomes in their regions. It reinforces the arguments of those who believe that moving more births into facilities with better trained birth attendants represents the best chance to reduce the very high maternal and perinatal mortality rates in many low income countries. However, as we await this transition, the survey also highlights some areas where training might improve outcomes for HBA-conducted deliveries—which still comprise most of the births that occur today in many resource-limited settings. For example, teaching uterine massage in the immediate post-partum period might reduce post-partum hemorrhage and lead to a concomitant decrease in maternal mortality, while training not to spank or shake newborns might prevent some neonatal injuries.

Until about 10 years ago, when there was little hope that large numbers of developing country home deliveries could be relocated to hospitals, many efforts were made to upgrade the skills of HBAs. In most studies, there appeared to be little improvement in pregnancy outcomes associated with this training. However, more recently, a study in Tibet found that community health workers could, in fact, be effectively trained to perform uterine massage, as well as in appropriate neonatal resuscitation techniques [17]. Results from a recent Global Network trial suggest that training HBAs in newborn resuscitation might reduce the number of births formerly characterized as stillbirths [10]. Examining the results of the controlled trials of traditional birth attendant training, the authors of a Cochrane Review summarized the limited data available by stating that there is potential to reduce perinatal mortality through HBA training, especially when done in conjunction with building a stronger linkage to the health system [14,17]. It is clear, however, that these reductions do not typically reach levels of perinatal mortality achievable with high quality hospital care for the mother and infant. Treating many of the conditions that result in the deaths of mothers, fetuses and many newborns (e.g. obstructed labor, placental abruption, preeclampsia/eclampsia, intrapartum asphyxia) usually requires high levels of diagnostic skills, and treatments typically available in hospitals including cesarean section and blood transfusion [18]. These interventions, now collectively known as emergency obstetric and newborn care or EmONC, when made available in hospitals to populations who formerly delivered at home, have been associated with substantial improvements in pregnancy outcomes. The low levels of literacy and formal schooling found in most HBAs in our study, suggest that even with additional training, many HBAs will not be able to acquire the skills to perform high level obstetric and newborn care.

Another approach might be to train skilled birth attendants such as nurse midwives with the intent that they practice in the communities performing home births. If strongly linked to the health system with rapid transport of patients with obstetric complications to a facility able to deal the complications, decreases in maternal and perinatal mortality will likely occur. However, in many low income countries, transportation even across short distances is a problem, and without timely access to a facility for delivery, important decreases in mortality with this approach are not likely to occur.

## Conclusions

Over the years, there have been extensive discussions regarding the appropriate role for HBAs in providing perinatal care, especially for women delivering at home. Since in many parts of the world there are no immediate alternatives to HBAs performing home deliveries, in the short term, it appears reasonable to provide those conducting home births with training in safe birthing practices and the recognition of life-threatening complications, while at the same time, linking them as strongly as possible with the formal health system. In the longer term, or presently in locations where hospitals can absorb the care for women currently delivering at home, an appropriate role for many HBAs might be to help direct pregnant women and their newborns, and especially those with complications, to facilities able to care for them. This approach seems appropriate since, despite their low level of education and medical skills, many of the HBAs are valued members of their communities and are often the local source of information and advice regarding childbirth. As countries or regions move more of their deliveries into hospitals, rather than shunting the HBAs aside, it seems appropriate whenever possible to value the stature of the HBAs in their communities and with appropriate training, explore a role for the HBAs in providing life saving care to pregnant women and their newborns. Incorporating HBAs into the formal health system as pregnancy advisers, as is currently being done in parts of India, seems like an experiment worth undertaking.

## Competing interests

The authors declare that they have no competing interests.

## Authors’ contributions

AG, EMcC and RG conceived of the study, developed the initial study instrument and wrote the majority of the draft, and reviewed and finalized the manuscript. EC, AP, OP, AT, FE, SG participated in the study design, reviewed the study instruments, oversaw the study implementation at their sites and reviewed the manuscript. KMH, LLW, MK-T, CB, WAC, EAL, PLH, SB reviewed the study protocol and participated in the study design, oversaw data collection and edited and reviewed the final manuscript. RW performed data quality monitoring, data edits and the statistical analyses and edited and reviewed the final manuscript. All authors read and approved the final manuscript.

## Funding

The study was funded by the Bill & Melinda Gates Foundation and the *Eunice Kennedy Shriver* National Institute of Child Health and Human Development. The investigators were solely responsible for the design and implementation of the study, without influence by the funding agencies (uoi HD040636).

## Pre-publication history

The pre-publication history for this paper can be accessed here:

http://www.biomedcentral.com/1471-2393/12/34/prepub
